# The impact of chemical pollution across major life transitions: a meta-analysis on oxidative stress in amphibians

**DOI:** 10.1098/rspb.2024.1536

**Published:** 2024-08-28

**Authors:** Colette Martin, Pablo Capilla-Lasheras, Pat Monaghan, Pablo Burraco

**Affiliations:** ^1^ School of Biodiversity, One Health and Veterinary Medicine, University of Glasgow, Glasgow G12 8QQ, UK; ^2^ Doñana Biological Station (CSIC), Seville 41092, Spain; ^3^ Zoological Institute, Technische Universität Braunschweig, Mendelssohnstraße 4, Braunschweig 38106, Germany; ^4^ Swiss Ornithological Institute, Bird Migration Unit, Seerose 1, Sempach 6204, Switzerland

**Keywords:** contamination, global change, life history, metamorphosis, oxidative damage, trade-offs

## Abstract

Among human actions threatening biodiversity, the release of anthropogenic chemical pollutants which have become ubiquitous in the environment, is a major concern. Chemical pollution can induce damage to macromolecules by causing the overproduction of reactive oxygen species, affecting the redox balance of animals. In species undergoing metamorphosis (i.e. the vast majority of the extant animal species), antioxidant responses to chemical pollution may differ between pre- and post-metamorphic stages. Here, we meta-analysed (*N *= 104 studies, *k = *2283 estimates) the impact of chemical pollution on redox balance across the three major amphibian life stages (embryo, tadpole, adult). Before metamorphosis, embryos did not experience any redox change while tadpoles activate their antioxidant pathways and do not show increased oxidative damage from pollutants. Tadpoles may have evolved stronger defences against pollutants to reach post-metamorphic life stages. In contrast, post-metamorphic individuals show only weak antioxidant responses and marked oxidative damage in lipids. The type of pollutant (i.e. organic versus inorganic) has contrasting effects across amphibian life stages. Our findings show a divergent evolution of the redox balance in response to pollutants across life transitions of metamorphosing amphibians, most probably a consequence of differences in the ecological and developmental processes of each life stage.

## 1. Introduction

Anthropogenic pollution is considered a major cause of biodiversity loss worldwide [1]. In particular, chemical organic and inorganic pollutants such as pesticides, metallic elements or pharmaceuticals are released daily into ecosystems through multiple sources, often resulting in novel and stressful conditions for wildlife [2]. The susceptibility of organisms to these pollutants could change over the course of their lifetime, which could be especially important for species with a life cycle that includes abrupt life transitions such as metamorphosis [3]. However, no study has systematically investigated to what extent anthropogenic chemical pollutants impact physiology over the life stages of metamorphosing species. Such an assessment would increase our understanding of how chemical pollution impacts species’ resilience and what the most vulnerable life stages are. This is essential knowledge for developing conservation actions and reducing biodiversity loss.

Reactive oxygen species (ROS) are unstable molecules generated from exogenous sources (such as pollution and UV radiation) or as by-products of cellular metabolic processes, with most produced during aerobic respiration in the mitochondria [4]. In low concentrations, ROS are critical for biological processes since they are involved in immune responses, detoxification and intracellular signalling [5,6]. However, exposure to ROS often causes cell damage, and when the accumulation of ROS overpasses the capacity of antioxidant enzymes to counteract them, a cellular oxidative stress state is induced [6,7]. Oxidative stress can damage essential biomolecules such as lipids, proteins or DNA, and finally lead to reductions in organismal health and life expectancy [7,8]. The physiological mechanisms that maintain the redox balance are thought to have a central systemic role and mediate life-history trade-offs, such as during adaptive growth or developmental responses which often include elevated ROS production [9–12].

The antioxidant system is highly conserved across taxa and consists of a wide range of enzymatic and non-enzymatic components that work synergistically to control ROS production and achieve redox homeostasis [13]. The first line of defence in response to oxidative damage involves the endogenously produced enzymatic scavengers such as superoxide dismutase, catalase, glutathione peroxidase or glutathione reductase [14]. The second line of defence involves scavenging non-enzymatic antioxidants with a low molecular weight that allows detoxifying ROS located in cellular areas where large enzymes cannot reach [13]. The tripeptide-reduced glutathione (GSH) is the most abundant non-enzymatic molecule in animal cells and can directly scavenge ROS or work in conjunction with antioxidant enzymes [5]. The redox balance is therefore set by the action of enzymatic and non-enzymatic antioxidant pathways [7]. Finally, some substances are produced under a scenario of oxidative stress as a result of damage to essential biomolecules. Malondialdehyde (an end product of the peroxidation of polyunsaturated fatty acids) is the most common marker of oxidative damage in the lipids of the cell membrane [15,16]. Both the production of ROS and the capacity to neutralize them can be influenced by chemical pollutants via diverse pathways [15,16].

Chemical pollutants exhibit distinct and complex modes of action and biological effects [15]. Pesticides, widely used in agriculture to protect various crops from pests, frequently contaminate surface water in freshwater ecosystems [17]. They disrupt cellular homeostasis by interacting with the mechanisms that regulate ROS production, resulting in oxidative stress [17,18]. Notably, pesticides affect the Keap1/Nrf2/ARE pathway, which plays a pivotal role in controlling the expression of antioxidant enzymes [17,18]. Moreover, the mitochondria play a critical role in energy production and cell death, and pesticides inhibit mitochondrial complexes, impairing mitochondrial function and increasing ROS production [17]. Heavy metals released from industrial waste accumulate in the bodies of aquatic organisms and interact with their organelles, posing significant health risks [19]. Metals like cadmium and lead specifically target the mitochondria, altering the permeability of the mitochondrial membrane. This leads to an increase in ROS and inhibits the activity of antioxidant enzymes, resulting in elevated oxidative damage and increased cell death [19]. A mixture of pharmaceuticals and personal care products (PCPs) typically enters aquatic ecosystems through wastewater discharge due to incomplete removal in water treatment plants [20]. Pharmaceuticals encompass a wide variety of drugs, many of which are biologically active and are capable of inducing physiological effects on freshwater organisms at low doses [20]. Prominent pollutants, including anti-inflammatory and antibiotic drugs, inhibit the antioxidant machinery, increasing susceptibility to oxidative damage [21,22]. Similarly, PCPs such as cosmetics and sunscreens contain chemicals and nanomaterials that persist in the environment [23]. Chemicals in these products, including antimicrobials and UV filters, are lipophilic and permeate membranes, interfering with enzymatic activities and antioxidant responses [23].

The effect of anthropogenic chemical pollutants on an organism’s redox balance could be linked to an organism’s antioxidant capability as well as its life mode. Both of these can change across an individual’s ontogeny. This is expected to be particularly relevant across the life cycle of species undergoing metamorphosis, a major life transition undergone by approx. 80% of existing animal species [3]. Metamorphosing species normally show three remarkably different stages (i.e. embryo, larva and adult) with contrasting phenotypic and physiological characteristics. Among vertebrates, amphibians are an ideal group in which to study the impact of chemical pollution on the redox balance across contrasting life stages. The life cycle of most amphibians, particularly of anurans, includes an embryo that hatches into a fish-like tadpole that abruptly develops into a tetrapod juvenile through metamorphosis [24]. Both embryos and tadpoles often have a highly permeable external surface/skin, and their habitat is commonly restricted to the aquatic environment [25,26]. In contrast, post-metamorphic individuals normally develop less permeable skin and, although they often rely on waterbodies for breeding, can normally inhabit the terrestrial environment [25]. Hence, the impact of substances released to water bodies, such as pollutants, is expected to vary across amphibian life stages, with the embryonic and larval stages potentially being the most vulnerable [26]. Amphibians are the most threatened vertebrate group, and chemical pollution is thought to be an important factor in their decline [27]. Understanding the extent to which pollutants adversely affect the amphibian redox state at different life stages will add important knowledge for the conservation of these and other metamorphosing species.

During the last three decades, a considerable number of studies have investigated the effect of pollutants on the amphibian redox balance either at their pre- or post-metamorphic stages. Making use of such information, we evaluate the impact of anthropogenic chemical pollution (e.g. organic and inorganic pollutants) on different aspects of the redox machinery (enzymatic and non-enzymatic antioxidant responses, and oxidative damage in lipids) in amphibians across their three major life stages (embryo, tadpole, adult). We carried out a systematic literature review and meta-analysis to assess whether the impact of chemical pollutants on the amphibian redox balance varies across amphibian life stages. We predicted that exposure to pollutants will increase ROS production in both pre- and post-metamorphic life stages. However, the external surface of amphibian embryos and tadpoles is highly permeable and, therefore, can easily absorb water-borne chemicals, and they are unable to evade aquatic pollutants. Therefore, we predicted they will have evolved a high antioxidant buffering mechanism, which will provide more protection against pollutants. Consequently, since post-metamorphic amphibians are often less exposed to chemical pollutants due to their impermeable skin and ability to evade unfavourable aquatic conditions by utilizing terrestrial habitats [25], we predicted a lower antioxidant capacity which would result in high oxidative damage in the presence of chemical pollution. Finally, since organic and inorganic pollutants have different modes of action, we expected that the pollutant type will lead to different consequences on the amphibian redox balance across life stages.

## 2. Methods

### (a) Literature review

Studies of the effects of anthropogenic chemical pollutants on the redox balance of amphibians were identified via relevant database searches conducted between the third and the tenth of March 2024. We used the search string, (‘oxidative stress’ OR ‘ROS’ OR ‘redox’ OR ‘antioxidant’) AND (‘amphibian’ OR ‘frog’ OR ‘toad’ OR ‘anuran’ OR ‘metamorphosis’) AND (‘pollution’ OR ‘chemical pollutants’ OR ‘water pollutants’ OR ‘Chemical toxicity’ OR ‘contaminant’ OR ‘contamination’ OR ‘metal’ OR ‘pesticide’ OR ‘fungicide’ OR ‘herbicide’ OR ‘insecticide’ OR ‘Pharmaceuticals’). We performed the search on EMBASE (Ovid), MEDLINE (EBSCOhost), PubMed, Scopus and Web of Science [28]. The search string was entered in the respective fields for each database: ‘Keyword’ in EMBASE (Ovid), ‘Article title, Abstract, Keywords’ in Scopus, ‘Topic’ in Web of Science, ‘All Fields’ in PubMed and the ‘Find any of my search terms’ search mode in MEDLINE (EBSCOhost). We read the title and abstract of studies published between 1974 (the earliest year with published data on the meta-analysed topic) and 2024 (the year the search was conducted), and we assessed whether studies contained suitable information for our meta-analysis (details below). We identified 1376 studies via the database searches above, plus nine additional studies that were identified from the reference list of screened studies (electronic supplementary material, figure S1). After removing duplicates, 645 studies were screened by reading their title and abstract, and 240 studies were identified as potentially containing suitable information for the meta-analysis. These 240 studies were examined in detail to assess whether they contained information that met the inclusion criteria (see details below). Database searches, study screening and effect size extraction were all performed by one co-author (C.M.). Most of the data in the papers were presented graphically, and thus numerical data was obtained using the digitalizing software WebPlotDigitiser version 4.4 [29], which has been shown to be a valid and accurate method of data extraction for meta-analyses [30].

### (b) Criteria for inclusion

We were interested in meta-analysing the effects of different pollutants on oxidative stress in amphibians based on controlled laboratory conditions. Therefore, we only included experimental studies that reported: (i) mean oxidative stress values, variation (standard deviation or standard error) and sample sizes (i.e. number of individuals) for control (i.e. non-exposed to pollutants) and treatment groups (i.e. exposed to pollutants); (ii) one of the following indices markers of the oxidative balance: superoxide dismutase, glutathione peroxidase, catalase, glutathione reductase, glutathione S-transferase (i.e. enzymatic biomarkers), GSH (i.e. non-enzymatic biomarker) or malondialdehyde (i.e. a marker of oxidative damage in lipids); (iii) the developmental stage (embryos, tadpoles or post-metamorphic) in which the effect of pollutants was tested. Additionally, we only included effect sizes from studies that tested one pollutant at a time (i.e. studies not testing the effect of a pollutant in combination with another factor) and studies conducted under laboratory conditions. The full list of pollutants included in this study can be found in electronic supplementary material, table S1. The articles included in this study were not evaluated for study quality during the screening process [28].

After assessing for inclusion (electronic supplementary material, figure S1), we extracted 2283 effect sizes from 104 studies [21–23,31–127]. These 104 studies contained information from 35 amphibian species relatively well distributed across the globe and the amphibian phylogeny (electronic supplementary material, figures S2 and S3). All these species are anurans with a life cycle including an embryo, tadpole and adult (post-metamorphic) stage, and the dataset respectively included 169, 1148 and 966 oxidative stress estimates from these three life stages (see electronic supplementary material, table S2). The 10 pollutant classes included in this study (fungicides, herbicides, metallic elements, nanoparticles, PCPs, pesticides, pharmaceuticals, polychlorinated biphenyls, polycyclic aromatic and polyfluoroalkyl chemicals) were broadly grouped as an ‘organic compound’ or an ‘inorganic compound’ for analysis (electronic supplementary material, tables S1 and S2).

### (c) Meta-analytic effect sizes

We compiled the dataset, ran all analyses, and produced visualizations using R (v. 4.3.3; [128]). To assess the effects of different pollutants on the oxidative stress of amphibians, we computed the log response ratio (lnRR) [129]. We calculated lnRR and its associated sampling variance using the R function ‘escalc’ in the ‘metafor’ R package (v. 4.6-0; [130]). We calculated lnRR so that positive values meant higher values of a given oxidative stress biomarker in the treatment group (i.e. after the exposure to a pollutant) than in the control group (i.e. not exposed to a pollutant), and *vice versa* for negative lnRR values. When a given control group was compared with multiple treatment groups, we divided the sample size of the control group by as many comparisons the control group was used for, and we used this adjusted sample size to calculate lnRR and its sampling variance (98 observations were removed due to a final sample size lower than one). The reported control or treatment standard deviation was zero for 46 observations. These data were retained in the analysis (assigning their standard deviation to 0.01) after checking that their reported s.d. was correct.

### (d) Meta-analysis

To assess how oxidative stress markers are affected by chemical pollution, we ran a phylogenetic multilevel (intercept-only) meta-analysis and meta-regressions. These models included three random intercept effects: publication identity, phylogeny and species identity, the latter to capture among-species variation not explained by phylogeny. Additionally, an observation identity random term was included to capture variation in effect sizes within studies. For intercept-only models, we estimated total heterogeneity (*I* ^2^_total_) [131] and the amount of variation explained by each random term as implemented in the R function ‘i2_ ml’ (‘orchaRd’ R package v. 2.0 [132]). For meta-regressions, we report on the proportion of variation explained by each moderator as calculated by the R function ‘r2_ ml’ (‘orchaRd’ R package v. 2.0 [132]).

To understand the overall effect of chemical pollution on the redox state, we ran two models. First, we ran an intercept-only model that contained the random effect structure explained above and no moderators. Second, we ran a meta-regression, including the random effect structure presented above and redox biomarker (‘enzymatic antioxidant’, ‘damage’ and ‘non-enzymatic antioxidant’) as a moderator. To understand the effect of chemical pollution across amphibian life stages, we repeated the meta-regression model presented above (i.e. including the ‘redox component’ as a moderator) for embryo, tadpole and adult life stages separately. To understand the effect of different chemical pollutants across amphibian life stages, we carried out meta-regressions for each type of pollutant (i.e. organic and inorganic) for embryo, tadpole and adult life stages separately. These models included the random effect structure presented above. Finally, we also examined the effect of pollutants on the amphibian redox balance in different tissues types and found that the effect was similar across tissues (electronic supplementary material, figure S4).

### (e) Phylogenies

To control for phylogenetic history, we extracted phylogenetic trees from Open Tree of Life [133,134], accessed via the R package ‘rotl’ (v. 3.1.0; [135]). Tree branch length was calculated following [136] and we generated a phylogenetic correlation matrix that was included in all our models. We assessed the phylogenetic importance in our meta-models calculating the proportion of variation in lnRR explained by the phylogeny (*I* ^2^_phylogeny_) [137].

### (f) Publication bias

We tested small-study effects and time-lag effects following Nakagawa *et al.* [138] by running two additional multilevel meta-analytic models of lnRR. Each of these models included, as a single moderator, either the square root of the inverse of the effective sample size or the mean-centred year of study publication [138,139].

## 3. Results

### (a) Overall effect of pollution on the redox balance of amphibians

**Figure 1.**
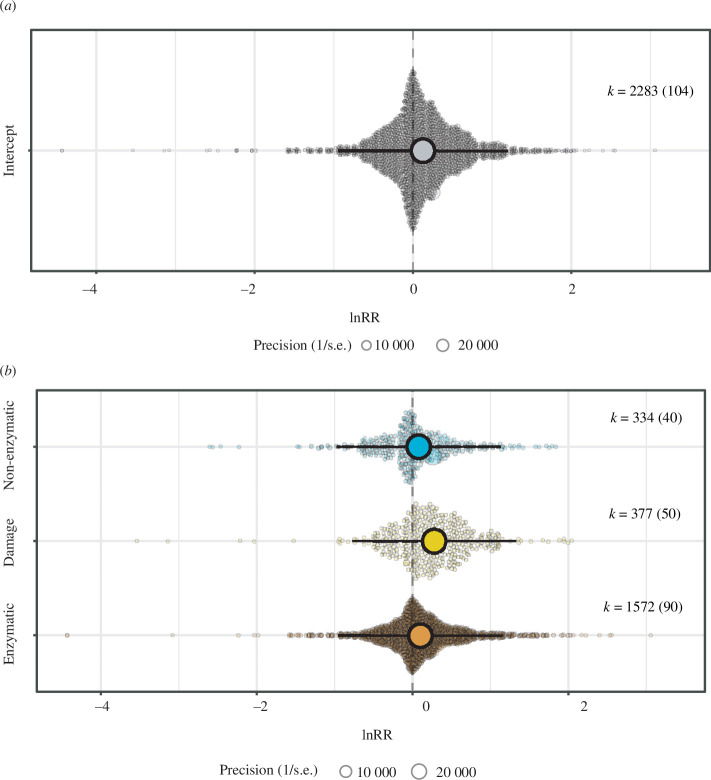
Orchard plots showing the overall effect of pollutant exposure on (*a*) the redox machinery (i.e. pooling all the collected estimates), and (*b*) enzymatic and non-enzymatic parameters, and indicators of oxidative damage in lipids of amphibian species undergoing metamorphosis. Coloured dots show means, a thick whisker 95% confidence interval and a thin whisker 95% precision interval. The precision (1/s.e.) of each study is represented in the background with scaled grey dots: the bigger the point, the bigger the higher the precision. Positive values on the *x*-axis represent a higher level of a given parameter in response to a pollutant.

An initial meta-analysis (i.e., intercept-only model) including all the oxidative stress parameters and life stages showed that experimental exposure to pollutants increased the levels of redox balance components (i.e. enzymatic and non-enzymatic antioxidants, and lipid damage) by 13% compared with control conditions (model intercept [95% confidence interval; ‘95% CI’ hereafter] = 0.126 [0.002, 0.251]; figure 1*a*). The total heterogeneity of this model was high (*I* ^2^_total_ = 99.98), with 2.72% and 2.75% of it explained by species and phylogeny, respectively, and 26.85% explained by among-study differences.

Exposure to pollutants increased the levels of all studied redox markers, with 95% CI for model estimates not overlapping zero for lipid damage (estimate [95% CI] = 0.278 [0.152, 0.404]; figure 1), and slightly overlapping zero for enzymatic antioxidants (estimate [95% CI] = 0.100 [−0.018, 0.217]; figure 1*b*) and non-enzymatic antioxidants (estimate [95% CI] = 0.078 [−0.050, 0.207]; figure 1*b*).

### (b) Effect of chemical pollution across amphibian life stages

**Figure 2.**
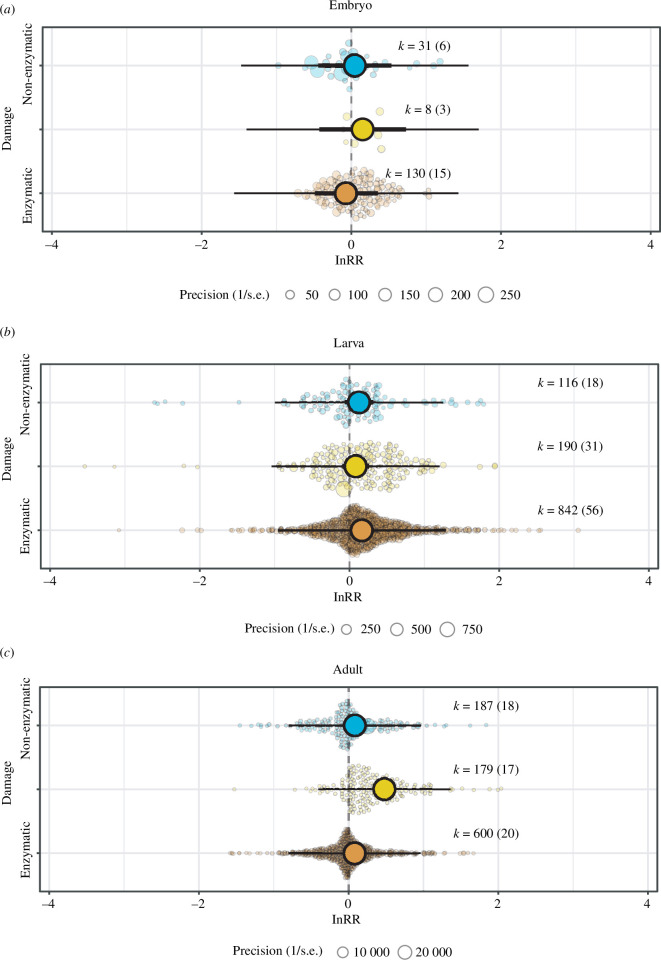
Orchard plots showing the effect of pollutants on the redox machinery (i.e. enzymatic and non-enzymatic components, and indicator of oxidative damage in lipids) of amphibian embryos (*a*), tadpoles (*b*) and adults (*c*). Coloured dots show means, a thick whisker 95% confidence interval and a thin whisker 95% precision interval. The precision (1/s.e.) of each study is represented in the background with scaled grey dots: the bigger the point, the bigger the higher the precision. Positive values on the *x*-axis represent a higher level of a given parameter in response to a pollutant.

Pollutants had a contrasting effect on the redox balance of embryos, tadpoles and adults. In embryos, pollutants did not increase the levels of the non-enzymatic antioxidants (estimate [95% CI] = 0.047 [−0.442, 0.535]; figure 2*a*), enzymatic antioxidants (estimate [95% CI] = −0.067 [−0.487, 0.352]; figure 2*a*) or lipid peroxidation (estimate [95% CI] = 0.151 [−0.428, 0.731]; figure 2*a*). The redox marker overall explained 0.68% of the heterogeneity in redox response to pollutants in embryos (i.e. *r* ^2^_marginal_ = 0.68%). In tadpoles, pollutants increased to a similar extent the levels of the enzymatic and non-enzymatic antioxidants (estimate ‘enzymatic’ [95% CI] = 0.164 [0.002, 0.326; figure 2*b*] and estimate ‘non-enzymatic’ [95% CI] = 0.127 [−0.062, 0.317]; figure 2*b*). In contrast, the effect of pollutants on lipid peroxidation in tadpoles was very low (estimate [95% CI] = 0.082 [−0.096, 0.260]; figure 2). Redox markers explained 0.30% of the overall variation in redox response to pollutants in tadpoles (i.e. *r* ^2^_marginal_ = 0.30%). In adults, while pollutants had a weak effect both on the enzymatic (estimate [95% CI] = 0.081 [−0.051, 0.212]; figure 2*c*) and non-enzymatic antioxidants (estimate [95% CI] = 0.083 [−0.056, 0.222]; figure 2*c*), lipid peroxidation levels were substantially increased (estimate [95% CI] = 0.478 [0.338, 0.618]; figure 2*c*). Redox markers explained 10.72% of the overall variation in redox response to pollutants in adults (i.e. *r* ^2^_marginal_ = 10.72%).

### (c) Effect of type of pollutants on the amphibian redox state

**Figure 3.**
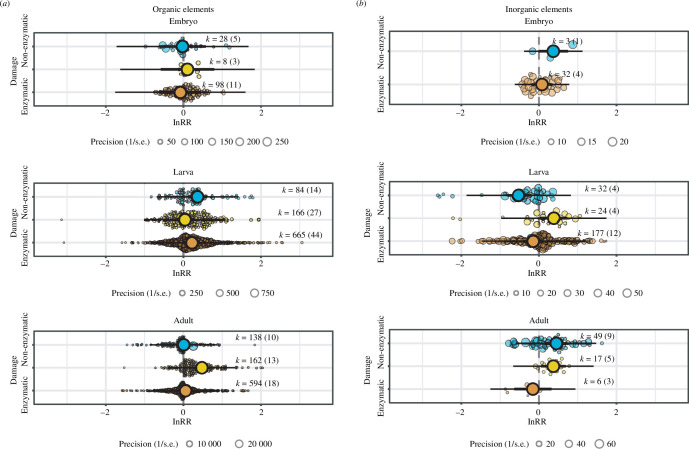
Orchard plots showing the effect of (*a*) organic and (*b*) inorganic pollutants, on the redox machinery (i.e. enzymatic and non-enzymatic components, and indicator of oxidative damage in lipids) of amphibian embryos, tadpoles, and adults. Coloured dots show means, a thick whisker 95% confidence interval and a thin whisker 95% precision interval. The precision (1/s.e.) of each study is represented in the background with scaled grey dots: the bigger the point, the bigger the higher the precision. Positive values on the *x*-axis represent a higher level of a given parameter in response to a pollutant.

We first investigated the effect of pollutants according to whether they were organic or inorganic contaminants. Inorganic pollutants only had subtle effects on the redox balance of embryos (figure 3*a*). In contrast, organic pollutants increased both the enzymatic and non-enzymatic response in tadpoles with no effect on their lipid peroxidation levels (‘enzymatic’ estimate [95% CI] = 0.221 [0.010, 0.431], ‘non-enzymatic’ estimate [95% CI] = 0.365 [0.127, 0.602]; figure 3*a*) and, in adults, induced lipid peroxidation but no antioxidant elevation (‘indicator’ estimate [95% CI] = 0.477 [0.312, 0.641], figure 3*a*). The available information for inorganic pollutants is much less; these only had weak effects on the non-enzymatic and enzymatic components of the redox machinery of embryos and adults, respectively (figure 3*b*).

### (d) Publication bias

We did not detect small-study effects in our dataset (estimate for the square root of the inverse of the effective sample size [95% CI] = 0.030 [−0.234, 0.294]), with the overall model intercept after correcting for effective sample size being similar to the overall model estimate without this correction (unbiased estimate [95% CI] = 0.108 [−0.091, 0.307]; overall model intercept presented above = 0.126 [0.002, 0.251]). We also did not detect time-lag effects (estimate [95% CI] = −0.004 [−0.015, 0.008]).

## 4. Discussion

Human activities lead to the release of chemical pollutants into ecosystems, which is threatening biodiversity across the globe. Our meta-analysis provides a comprehensive assessment of the impact of chemical pollution on amphibian redox state. Overall, exposure to chemical pollutants increases levels of the key redox biomarkers, indicative of endogenous responses to oxidative stress. However, this effect is life-stage-dependent: while embryos do not experience any variation in terms of their redox components, tadpoles exhibit increased antioxidant responses and, overall, experience no elevation in oxidative damage to lipids, and adults show a lack of antioxidant responses and marked lipid peroxidation. The type of pollutant also has life-stage-specific effects on the redox components of amphibians, although the literature is biased towards the effect of specific organic pollutants such as pesticides and herbicides.

The life cycle of most extant animal species includes some form of metamorphosis. This major life transition not only includes conspicuous developmental transformations but also changes in nutrition, niche use and behaviour [3]. In metamorphosing amphibians, embryos and tadpoles have a permeable external surface and are often restricted to water bodies, while post-metamorphic individuals generally have protective skin and the ability to explore both the aquatic and terrestrial environments. In our meta-analysis, we found no effect of chemical pollutants on the redox state of embryos. The lack of antioxidant response might be explained by the allocation of resources to somatic maintenance and development at the expense of experiencing a low antioxidant capacity, however, this does not explain the absence of oxidative damage which may be due to the protective role of the embryo jelly. Embryos are the least represented group in our meta-analysis and thus further information is still desirable. Our findings also show that the tadpole antioxidant machinery is upregulated against chemical pollutants, which likely explains the lack of oxidative damage. This pattern contrasts with that observed in post-metamorphic amphibians faced with pollutants, which show negligible antioxidant responses and marked oxidative damage. Differences in ecological and developmental processes across amphibian life stages may have driven a divergent ability to cope with variations in environmental pollution. In other words, the developmental processes experienced at each life stage, the likelihood of being exposed to chemical pollutants and/or their lifestyle may have led to a divergent evolution of antioxidant responses across life stages of metamorphosing species [140,141]. Oxidative stress is also thought to play a major role in modulating life-history trade-offs, including the balance between important aspects of fitness such as growth and survival [9]. In metamorphosing organisms challenged with pollutants, avoiding an oxidative stress state could allow pre-metamorphic individuals to reach metamorphosis with a body size large enough to reduce post-metamorphic mortality rates [142,143]. At least two meta-analyses have suggested that animals show stronger responses to stress at early stages than later in life, which could be a mechanism to avoid developmental impairment and negative carry-over effects [144,145]. However, the evolution of redox responses across an organism’s lifespan may be constrained by its maintaining cost and its relationship with life histories, including phenotypic plasticity [146,147]. Further empirical and comparative studies will disentangle whether life-stage-dependent redox responses are context- and/or taxa-specific in species undergoing metamorphosis.

Chemical pollution commonly leads to negative consequences for wildlife. The mechanisms behind this process have been well studied in some taxonomic groups responding to specific pollutants. Although the mechanisms of action can differ between pollutants, both carbon- and non-carbon-based pollutants often cause metabolic and endocrine dysfunctions that can finally result in molecular damage such as oxidative stress [148–150]. In amphibians with complex life cycles, the role of the redox machinery in organisms coping with chemical pollution has been extensively investigated, hence allowing to meta-analyse life-stage-specific effects of such pollutants. Our study shows that, overall, chemical pollutants impact the redox balance of amphibians hence altering oxidative eustress (i.e. the presence of low antioxidant levels found during normal metabolism maintenance) and leading to oxidative distress (i.e. impaired eustress) [6,151]. These effects vary depending on the life stage and the type of pollutant. It should be noted, however, that most of the available data come from studies on the effect of pesticides conducted both in tadpoles and adults, and herbicides in tadpoles (both types are organic pollutants). With a global production of two million tonnes, chemical pollutants are ubiquitous in the environment and often enter aquatic ecosystems, posing a major threat to semi-aquatic amphibians [152]. The impact of these pollutants on the amphibian redox balance that we report here might explain, at least partly, the decline of amphibian populations associated with chemical pollution [153]. Our study highlights the need for further research on the impact of other chemical pollutants such as metallic elements or other inorganic compounds on the redox balance of amphibians, for which the available information is still scarce. In this line, the impact of emerging pollutants (e.g. microplastics) on the amphibian redox performance is still virtually unknown. More experimental work to test the effect of these and other pollutants across all life stages will be needed to better understand the real-world impact of chemical pollution on metamorphosing animals. This contrasts with the broad test of those and other pollutants in behavioural studies [154]. Also, our meta-analysis only includes studies using a single pollutant (the most abundant throughout the literature) and experiments combining several pollutants with different characteristics (e.g. chemicals with light or noise pollution) are still scarce, however, they will improve our understanding of the wildlife responses to anthropic disturbed.

## 5. Conclusions

Our meta-analysis shows that the effect of chemical pollution on the redox balance of amphibians varies across the three major life stages of metamorphosing amphibians. While embryos do not alter any of the studied redox components, tadpoles induce antioxidant responses, but avoid oxidative damage, and adults show a lack of antioxidant response but pay an oxidative cost in terms of increased lipid peroxidation. Our study also shows that the type of pollutant can shape the amphibian redox status, which seems to be life-stage-dependent. Future studies will provide insights into the response to pollutants of different over the developmental trajectory of species undergoing metamorphosis. Experiments designed specifically to examine the link between chemical pollutants, the redox balance and life histories of metamorphosing organisms are needed.

## Data Availability

All R scripts and datasets needed to reproduce the analyses presented in this paper are now available at [155]. Supplementary material is available online [156].
